# Quality Assurance, Metrics, and Improving Standards in Rectal Cancer Surgery in the United States

**DOI:** 10.3389/fonc.2020.00655

**Published:** 2020-04-29

**Authors:** Zhaomin Xu, Fergal J. Fleming

**Affiliations:** Surgical Health Outcomes and Research Enterprise (SHORE), Division of Colorectal Surgery, Department of Surgery, University of Rochester Medical Center, Rochester, NY, United States

**Keywords:** colorectal cancer, colorectal neoplasms, quality improvement, surgical outcomes, patient-reported outcomes

## Abstract

Rectal cancer surgery has seen significant improvement in recent years. This has been possible in part due to focus on surgeon education and training, specific surgical quality metrics, and longitudinal tracking of data through the use of registries. In countries that have implemented such efforts, data has shown significant improvement in outcomes. However, there continues to be significant variation in rectal cancer outcomes and practices worldwide. Just within the United States, county level mortality rates from rectal cancer range from 8–15 per 100,000 to 38–59 per 100,000. In order to continue to improve rectal cancer patient outcomes, there needs to be evidence based guidelines and standards centered around the framework of structure, process, and outcomes. In addition, there must be a feedback system by which programs can continually assess their performance. Obtaining evidence for specific standards and measures can be challenging and requires analyzing available data and literature, some of which may be conflicting. This article evaluates the evolution of metrics and standards used for quality improvement in rectal cancer and ongoing efforts to further improve patient outcomes.

## Introduction

Rectal cancer continues to be a public health concern with 40,000 newly diagnosed cases annually in the United States (US) (1). Worldwide, colorectal cancer is the third most commonly diagnosed cancer and the fourth leading cause of death with 1.4 million new cases and almost 700,000 deaths in 2012 (2). Rectal cancer outcomes depend heavily upon the quality of surgical resection. Thanks to a concerted effort to improve rectal cancer resections, spearheaded by the concept of total mesorectal excision (TME) pioneered by MacFarlane et al. (3), there have been great strides made in the improvement of rectal cancer outcomes worldwide. However, there continues to be substantial variation in the outcome of rectal cancer patients. Therefore, the question remains of how to continue the trend of improving outcomes and decreasing variation.

The consensus is that the road to improving outcomes involves the capture of quality measurements. This has led to considerable debate and discussion about which quality measures best reflect surgical quality for rectal cancer. The National Quality Forum established four criteria for assessment of a measure for endorsement (4):

(1) Importance: A measure must be important to measure and report where the most impact can be made on healthcare quality.
(2) Usability and Relevance: A measure must be understandable and useful by intended users to improve quality.
(3) Scientifically Acceptable: A measure must produce consistent and credible results.
(4) Feasibility: The measure must be driven by data that is readily available and can be retrieved.

Quality measures can be categorized as either involving structure, process, or outcomes, based on the Donabedian classification [(5); Table 1]. Structural measures are the organizational features associated with delivery of care. Process measures refer to the care directly received by patients. Outcomes refer to results of treatment. Many single measures within the realm of these categories have been investigated for rectal cancer, but there is increasing evidence that for meaningful long term improvement, we need clinically relevant quality measures that span multiple phases of care (7). To achieve this, the National Accreditation Program for Rectal Cancer (NAPRC) was developed through a collaboration between the Optimizing the Surgical Treatment of Rectal Cancer (OSTRiCh) Consortium and the Commission on Cancer (CoC) of the American College of Surgeons (8). NAPRC proposes a set of standards pertaining to program structure and process of patient care, as well as performance indicators for the evaluation of outcomes. These standards were based on data supporting measures that can individually be classified in one of Donabedian's categories.

**Table 1:** Quality measures based on the Donabedian classification.

	**Structural**	**Process**	**Outcomes**
Examples	• Hospital and surgeon Volume • Hospital accreditation and certification • Care Fragmentation	• Guideline adherent treatment • Appropriate neoadjuvant administration • Staging and carcinoembryonic antigen (CEA) • Surgical quality (i.e., circumferential resection margin, lymph node yield) • Appropriate adjuvant therapy administration • Multidisciplinary team meetings	• Morbidity and mortality • Patient reported outcomes • Quality of life measures
Pros	• Easy and inexpensive to measure • Robust associations	• Easily tracked for quality improvement • Easy to act upon • Reflects direct care received by patients • Robust associations	• Easy interpretability for stakeholders • Measuring alone may improve outcomes
Cons	• Usually proxies for other measures • Difficult to act upon	• Difficult to determine measures for specific procedures	• Small sample sizes results in event rates that are too low to measure • National data may lack granularity for control of confounding • Need procedure specific measures

*Adapted from Birkmeyer et al. (6)*.

## Quality Measures

One of the driving factors for development of rectal cancer quality measures in the United States has been the data and experience published out of Europe. Efforts made by Norway included the establishment of national consensus for rectal cancer management through the development of guidelines for chemotherapy/radiation therapy, preoperative imaging and staging, pathologic examination, and multidisciplinary workshops held to educate providers on TME surgery technique. A Norwegian Rectal Cancer Registry was established in order to monitor outcomes and feedback outcomes to institutions. With these efforts, they were able to improve 5-year relative survival by 9% and decrease 5-year local recurrence by 9.5% over the course of 10 years (9). In the United Kingdom (UK), the Calman-Hine Report had shown that the UK lagged behind other European countries significantly in rectal cancer outcomes (10). In order to close this gap, the UK formulated the National Health Services Cancer Plan, which emphasized centers of excellence, defining protocols for specific phases of care, and collecting and monitoring outcomes. Through this concerted effort, they were able to improve 5-year overall survival from 48.8 to 61% over the course of 14 years (11).

Compared to European data, the US was found to lag behind in several metrics. Based on National Cancer Database (NCDB) data, the positive circumferential margin rate in the US was 17.2%, compared to 11% in the UK (12, 13). Rectal cancer patients in the US also had higher rates of colostomies compared to many European countries (14, 15). In addition, without any accreditation system, large numbers of rectal cancer patients were being treated a low volume centers (16).

From a structural standpoint, one of the major factors that has been found to impact rectal cancer outcomes is volume. Robust volume-outcome relationships have been consistently found in many complex oncologic surgical procedures (17–19). Aquina et al. found that in New York State, there has been a natural migration of rectal cancer resections to high volume providers and facilities. Higher rectal cancer resection volume has been shown to be associated with decreased rates of colostomy formation and 30-day mortality (20). National US data has shown similar results with high volume centers having higher rates of guideline adherent treatment and providing improved long-term overall survival compared to their low volume counterparts (21). Internationally, studies have also shown high volume centers to have improved outcomes including lower rates of positive circumferential resection margins, improved rates of adequate lymph node yield, lower operative mortality, and improved long-term survival (22–24). Another factor related to healthcare infrastructure that has recently been shown to have an impact on rectal cancer patient outcomes is care fragmentation. Justiniano et al. showed that rectal cancer resection patients who required readmission had a 2-fold decrease in survival if patients were readmitted to a different surgeon than the index surgeon, regardless of whether it was the index facility (25). Finally, one method used to improve quality and outcomes from a structural standpoint is accreditation. Two well-known accreditation programs for facilities providing cancer treatment are the American College of Surgeon's Commission on Cancer (CoC) and the National Cancer Institute's NCI-designation. CoC-approved centers typically offer more cancer-related services such as chemotherapy and radiation, and they also performed more cancer operations per year compared to non-CoC centers (26). Colorectal cancer patients treated at NCI-designated centers have previously been shown to have a 16% improvement in overall mortality compared to patients treated at non-NCI-designated centers (27). However, while the evidence for structural measures tends to be very robust, structural measures such as volume and care fragmentation are difficult to act upon without significant cost and time. In addition, it is difficult to determine whether structural measures are proxies for other measures that may be more readily acted upon.

Several process measures have been implicated in rectal cancer outcomes. Due to the complexity of rectal cancer treatment, including neoadjuvant and adjuvant therapy for locally advanced disease, there are many opportunities for improvements in process. US National data shows that compliance with National Comprehensive Cancer Network (NCCN) recommendation for neoadjuvant chemotherapy/radiation and adjuvant chemotherapy for locally advanced rectal cancer is poor. Only approximately 30% of locally advanced rectal cancers in the US received neoadjuvant therapy (28). Among those patients, only 32% of patients received the recommended sandwich therapy with adjuvant chemotherapy (29). In both cases, receipt of appropriate chemotherapy and radiation has been associated with improved survival. European data has also revealed issues with poor compliance to guidelines in colorectal cancer, though compliance appears to be better than the US. For example, Glynne-Jones et al. and Heins et al. found that 80–90% of T2 rectal cancer patients received neoadjuvant radiation therapy (30, 31). Another important aspect of rectal cancer care process is staging. Staging directly impacts the therapy that patients ultimately receive. Modern standard of care is to evaluate staging with MRI. In an analysis of national staging data for rectal cancer, there was evidence that a large number of patients (24%) were initially under-staged, which resulted in higher rates of positive circumferential resection margin (CRM) and worse 5-year overall survival (32). Another increasingly recognized process that appear to impact outcomes for colorectal cancer patients is multidisciplinary team meetings (MDT), particularly for advanced disease. Previous studies have shown that among advanced colorectal cancer patients, MDTs decreased the hazard to death by 35% (33). The benefit of process measures is that similar to structural measures, there tends to be robust evidence supporting their effectiveness. In addition, these measures are very actionable and easily tracked for quality improvement. The main weakness of process measures, particularly when applied to specific procedures, is that the measures may be too broad and therefore, may not result in any improved outcomes despite improved compliance.

Outcomes measurement is an obvious road to quality improvement and therefore has been used for years with the most common being the ACS National Surgical Quality Improvement Program (NSQIP). Most registries capture outcomes measures in one form or another. For example, NCDB and the Surveillance, Epidemiology, and End Results (SEER) program tracks patient's vital status. The most basic outcomes involve survival, either overall or disease specific. Beyond survival and recurrence, operative quality measures are also of significant interest. While there has been significant progress made in chemotherapeutics and optimizing radiation therapy, surgery remains the cornerstone of treatment for rectal cancer, and therefore is a crucial contributor to process measures. We showed with NCDB data that in the US, there was a 3.5-fold difference in the rate of an adequate lymph node yield of 12 among individual hospitals performing proctectomies for rectal cancer. This was again independently associated with increased 5-year mortality (34). Rickles et al. had found a positive CRM rate of 17.2% in the US. Interestingly, facility location was a predictor of CRM positivity, which indicates significant geographical variation in the quality of surgery (13). Another growing field of interest is the impact of surgical complications on long-term outcomes for cancer patients (35, 36). Wang et al. found in their meta-analysis that anastomotic leak after proctectomy for rectal cancer was associated with a 71% increase in the risk of local recurrence, a 67% increase in the risk of overall mortality, and a 30% increase in the risk of cancer-specific mortality (36). Outcomes measures are highly advantageous because of easy interpretability, which may improve buy-in from stakeholders. The largest weakness of outcomes measures is that the event rate of some measures may be too low to detect significant differences (6). Many investigators, including our group, have combated this by analyzing large national and statewide datasets, or even combining procedures that may be interpreted as having similar complexity, such as pancreaticoduodenectomies and esophagectomies. This obviously leads to a couple of issues. Large datasets likely lack enough granularity to control for all confounders, leading to difficulty in result interpretation. And aggregating other organ systems rely on assumptions that may or may not be true, again, leading to difficulty interpreting results. Another issue with outcomes measurement is whether the appropriate outcome is being measured. Traditionally, short-term perioperative mortality has been measured at the 30-day mark. However, there is evidence that by only assessing 30-day outcomes there is a significant burden of post-operative mortality that is being missed that could potentially be intervened upon. Byrne et al. found in their analysis of colorectal surgical patients that by examining 90-day mortality as opposed to 30-day mortality, they were able to find twice the number of hospitals that were high outliers for mortality (37). This study highlights the importance of choosing the right outcome measure in order to maximize quality improvement initiatives.

As discussed above, each individual measure has inherent weaknesses and strengths. Taken in isolation, individual measures are likely to miss opportunities for more significant improvement and synergies that may help decrease the overall cost of quality improvement. In many ways, NAPRC helps to bridge the gap between individual measures and having an evidence based quality improvement system that is able to evaluate the full spectrum of rectal cancer care delivery. The use of an accreditation system has been used to great effect by other countries such as Germany, where an accreditation system for colorectal cancer centers has existed since 2006 (38). NAPRC includes a set of 20 structure and process standards that include the development and maintenance of a multidisciplinary team, timing of treatment, standardized imaging and pathologic assessment, and a system of data submission and review. There are an additional 10 quality indicators, or outcomes measures, including many surgical quality indicators, which are meant to be collected and reported back to institutions for guidance of quality improvement [(39); Table 2]. There is ongoing work to help capture more measures nationally in order to better assess the impact of NAPRC, but based on currently available measures, Brady et al. found that only 28.1% of rectal cancer patients who underwent resection achieved all process measures and 56.3% achieved all outcomes measures (40). At the hospital level, Antunez et al. found that only 2.9% of hospitals in the US met all process measures. While the study was unable to find a direct impact on survival based on adherence to the available process measures, they did find that hospitals least likely to receive NAPRC accreditation were more likely to serve patients of lower socioeconomic position and have worse survival outcomes (41). In addition, New York state data shows that a model of centralization that limits proctectomies to only high volume centers could have a substantial impact on the travel distance of rectal cancer patients, particularly those in rural communities (21). This data hints at what is potentially a major gap in the current state of rectal cancer quality improvement: patient-centered outcomes.

**Table 2:** Proposed standards for the National Accreditation Program for Rectal Cancer (NAPRC).

**Structure and process standards**	**Quality indicators**
1. Must be Commission on Cancer accredited 2. Must have defined multidisciplinary team (MDT) 3. MDT members must attend at least 50% of rectal cancer MDT meetings every year 4, MDT meets at least twice a month 5. Must have a named MDT leader 6. Must have a rectal cancer program coordinator 7. Members of the MDT must complete NAPRC education modules 8. Rectal cancer patients must have confirmation of diagnosis by biopsy prior to initiating treatment at an accredited rectal cancer program 9. Patients must have appropriate staging prior to treatment 10. MRIs should be read by an MDT member radiologist and reported in a standardized report 11. A carcinoembryonic antigen level should be obtained prior to treatment 12. All patients must be discussed at MDT prior to initiation of treatment 13. Before initiation of treatment, the treatment plan should be provided to the primary care physician or referring provider 14. Patients should initiate treatment within 60 days of initial evaluation 15. Patients should be operated on by a member of the MDT and operative reports should be reported in a standardized fashion 16. Surgical specimens should be read by a member of the MDT and reports should followed a standardized format 17. Surgical specimens should be photographed 18. An individualized treatment outcome discussion should occur 4 weeks after definitive surgical treatment 19. Within 4 weeks of the treatment outcomes discussion, a summary report should be generated and provided to the primary care physician or referring provider 20. Patients eligible for adjuvant therapy should initiate therapy within 8 weeks of surgical resection	1. Abdominoperineal resection rate 2. Anastomotic leak rate 3. Reoperation rate 4. 30-day mortality rate after surgery 5. Involved circumferential margin rate 6. Involved distal resection margin rate 7. Mesorectal grade rate 8. Lymph node yield ≥12 rate 9. Local recurrence rate 10. 3-year disease-free survival rate

Many quality measures revolve around surgical measures because surgical measures tend to be easier to measure and there are robust administrative datasets that already collect the data necessary to monitor surgical measures. However, there are several aspects of rectal cancer care that lack meaningful quality measures. While we appreciate the important of multidisciplinary team meetings for rectal cancer patients, there are no measures for the quality of this process. This lack of assessment is a crucial gap as treatment decisions made during MDT meetings have significant impact on downstream outcomes. We also place significant emphasis on improving what we are doing already within the phases of rectal cancer care. However, a major piece that is missed if we only look at what we do is how patients feel about the impact of their treatments. While there are some measures available for capturing patient perspective, such as the Medicare Outcomes Survey and the Hospital Consumer Assessment of Healthcare Providers and Systems Survey (HCAHPS), they are not sensitive enough to provide data for surgical quality assessment. To address this gap, there are ongoing initiatives to develop validated questionnaires and surveys that measure many aspects of health such as severity of symptoms, health care experience, and quality of life (42). These measure are known as patient reported outcomes (PROs). PROs allow for early detection of distress in a patient, provides a valuable opportunity for patients to be heard, and allows for interventions to be directed toward specific symptoms (43). Thanks to new psychometric techniques and electronic platforms, much of this data can now be easily collected. To utilize this data, the ACS started a multiphase pilot study to assess the feasibility of collecting this data within the ACS NSQIP platform. The first phase assessed feasibility in breast, colorectal, endocrine, hepato-pancreato-biliary, thoracic, trauma, and general surgery patients (42). While previous discussions of measures falling within the Donabedian classification are more about standardization of care, PROs is an opportunity for more individualized care for cancer patients (44).

## Summary

There has been significant development in the improvement of rectal cancer quality measures. While the basis of quality measures can be categorized into structural, process, and outcomes measures, it is unlikely any one measure from any of these categories can singlehandedly improve the quality of rectal cancer care. Each measure has certain advantages and disadvantages. NAPRC is a more comprehensive program that includes measures from all phases of rectal cancer care to maximize quality improvement. Central to NAPRC, and the success of national programs instituted by other countries, is the ability of the program to track and feedback performance measures to institutions in order to facilitate continued advancement. The ultimate impact of NAPRC on rectal cancer care in the US remains to be seen, but what is becoming clear is that NAPRC alone will not be enough. With an increasing emphasis on patient centered care and outcomes, we can no longer only focus on what we, as providers do, but also what patients want and how they experience what we do to them. PROs is a strong step toward integrating this group of quality measures to our existing framework. The one certainty is that the development of a successful system for consistent quality improvement is crucial for the care of our rectal cancer patients.

## Author Contributions

ZX and FF contributed to the writing and editing of this manuscript.

## Conflict of Interest

FF receives royalties from UpToDate, Inc. The remaining author declares that the research was conducted in the absence of any commercial or financial relationships that could be construed as a potential conflict of interest. The reviewer KM declared a shared affiliation, with no collaboration, with the author FF to the handling editor at the time of review.
